# Spatially Stereotyped Microgliosis Tracks Synaptic Pathology in the Demyelinated Superior Colliculus

**DOI:** 10.21203/rs.3.rs-9829260/v1

**Published:** 2026-06-04

**Authors:** Jackson David McGrath, John Shultz, Maia Jin Classe, D. Ford Hannum, Sebastian Werneburg

**Affiliations:** Michigan Medicine; Michigan Medicine; Michigan Medicine; Michigan Medicine; Michigan Medicine

**Keywords:** Cuprizone, Multiple Sclerosis, Neuroinflammation, Superior colliculus, Synapse loss, Visual system

## Abstract

Visual impairment is one of the most common and clinically salient manifestations of Multiple Sclerosis (MS), yet pathology across visual system structures remains incompletely defined. Although MS pathology has been extensively studied in the optic nerve, lateral geniculate nucleus, and visual cortex, involvement of the superior colliculus (SC), a key hub for visual processing, has not been systematically investigated. Here, we combined human postmortem tissue analysis with functional assessment and spatial mapping in the MS-relevant cuprizone (CPZ) mouse model to define how demyelination and secondary injury are organized within the SC. Postmortem SC tissue from donors with MS revealed myelin loss, including focal demyelinated lesions. In mice, CPZ treatment impaired visual function and induced widespread demyelination across SC layers, without detectable neuronal cell loss or axonal degeneration. Although diffuse demyelination was accompanied by widespread microgliosis characteristic of CPZ, atlas-based mapping uncovered a previously unrecognized spatial organization: a discrete high-microgliosis compartment that emerged in every CPZ-treated SC with strikingly stereotyped location and shape. This compartment did not correspond to canonical SC maps and was not explained by baseline differences in microglia or myelin or by variability in demyelination severity following CPZ. Instead, regions with elevated microgliosis showed a marked increase in synaptic elimination, suggesting that secondary synaptic pathology may contribute to the spatial organization of microgliosis beyond diffuse myelin loss alone. Prolonged CPZ exposure expanded the compartment in a stereotyped pattern, whereas CPZ withdrawal produced spatially ordered partial resolution while leaving a persistent high-microgliosis core concurrent with partial visual recovery. Together, these findings identify the SC as an MS-relevant site of injury and establish the CPZ-treated SC as a reproducible *in vivo* model for studying spatially patterned microglial reactivity, synaptic pathology, and incomplete inflammatory resolution after demyelinating injury.

## INTRODUCTION

Multiple Sclerosis (MS) is a chronic disease of the central nervous system (CNS) in which demyelination, neuroinflammation, and neurodegenerative processes converge to disrupt neural circuit function and ultimately drive irreversible disability [18, 56, 78]. Visual dysfunction is among the most common and clinically salient manifestations of MS, often presenting early in the disease course [4, 15, 21, 57, 76]. Accordingly, the visual system has become a key model circuit in MS research, as its well-defined anatomy enables quantitative readouts to track dysfunction over time and, in some settings, relate early injury to subsequent disability [4, 15, 21, 57, 76].

Historically, visual symptoms in MS have been closely linked to inflammatory demyelination with associated axonal loss in white matter tracts, most prominently exemplified by optic neuritis—an acute inflammatory demyelinating attack of the optic nerve that typically presents with subacute, often painful, monocular vision loss [3, 21]. However, visual impairment is also common in the absence of a clinical history of optic neuritis, and the severity of dysfunction is often not explained by focal white matter lesion burden within the affected circuit [4, 8, 9, 19, 21]. These observations have motivated efforts to identify additional drivers of visual disability beyond white matter damage alone [9, 18, 56].

A growing body of evidence implicates gray matter pathology as a major contributor to disease severity and progression in MS [9, 18, 56, 78]. For instance, cortical abnormalities, including cortical atrophy and neuronal injury, have been repeatedly associated with clinical disability and progressive disease features [8, 9, 22, 23, 36, 39, 52]. Subcortical gray matter structures are also prominently affected [20, 55, 81, 82]. In particular, thalamic atrophy and microstructural abnormalities correlate with neurological impairment across disease stages [20, 55, 81, 82], supporting the concept that integrative gray matter hubs may be especially vulnerable in MS [42, 43, 81]. Within the visual system, pathology has been documented at multiple gray matter nodes, including the primary visual cortex and the lateral geniculate nucleus (LGN) of the visual thalamus [8, 9, 44, 49]. In the visual cortex, cortical thinning, lesion burden, and functional reorganization have been linked to visual processing deficits, whereas imaging and postmortem studies of the LGN have identified structural injury associated with visual impairment [4, 44, 49, 76]. Together, these findings indicate that visual disability in MS reflects distributed injury across multiple gray matter nodes within the visual pathway.

At the cellular level, synaptic pathology has emerged as an early and functionally relevant form of gray matter injury in MS and MS-relevant models [13, 17, 28, 32, 45, 46, 71, 76]. Human postmortem studies, synaptic PET imaging, and preclinical models have identified synaptic loss or dysfunction across several CNS regions [13, 17, 28, 32, 45, 46, 71, 76]. In the visual thalamus, our prior work showed that loss of retinal ganglion cell (RGC) synaptic inputs to the dorsal LGN can occur early, precede detectable neuroaxonal damage, and correlate with visual acuity decline in MS-relevant mouse models [76]. We further found that reactive microglia engulf RGC synapses, establishing microglia-associated synapse loss as a potential substrate of circuit dysfunction in MS [76]. Consistent with this concept, previous work using the cuprizone (CPZ) model of demyelination, one of the most widely used MS-relevant models [27, 66, 67], has reported altered subcortical visual circuit function together with synaptic loss and microgliosis in the LGN [2]. Thus, synaptic pathology and microglial reactivity are increasingly recognized as important features of MS-relevant gray matter injury. However, how these pathologies are spatially organized within affected visual system structures remains poorly understood.

The superior colliculus (SC) is a key yet comparatively understudied node in mammalian visual circuitry, particularly in the context of neurodegenerative disease [5, 6, 11, 77]. It integrates multisensory inputs to direct orienting and defensive behaviors and exhibits a conserved laminar organization, with superficial layers receiving dense, topographically mapped visual input and deeper layers integrating multimodal sensory and motor-related information to support visuomotor transformations [5, 11, 77]. Recent advances have provided a refined understanding of SC microcircuits, genetically defined cell types, and proposed functional subdivisions that map onto distinct behavioral repertoires, including orienting, arrest, and escape-like responses [5, 6, 11, 77]. This highly organized heterogeneous architecture makes the SC well-suited for asking whether demyelination-associated pathology follows established anatomical, functional, or connectivity-based maps, or instead reveals disease-associated spatial organization not predicted by canonical SC structure. Although the SC has been implicated in several neurological disorders characterized by disrupted visual processing and sensorimotor integration, including Parkinson’s disease, where altered SC-dependent visual responses have been reported in *de novo* patients [6, 47, 53, 59], systematic neuropathological characterization of the SC in MS or MS-relevant models has not been performed.

To address this gap, we integrated analysis of postmortem human SC tissue with functional testing and atlas-registered spatial mapping in the CPZ mouse model, which provides a controlled system to investigate CNS-intrinsic responses to oligodendrocyte loss and subsequent repair after toxin withdrawal [27, 66, 67]. Here, we identify SC demyelination in postmortem tissue from donors with MS and show that CPZ induces widespread SC demyelination and visual dysfunction in mice while preserving neuronal and axonal densities. Despite diffuse myelin loss and broad accompanying microglial activation, atlas mapping identified an unexpected, discrete, and highly stereotyped high-microgliosis (HG) compartment in all CPZ animals. Notably, this HG compartment did not correspond to established SC anatomical, functional, or connectivity-based maps and was not explained by local variation in demyelination severity. Instead, the HG compartment coincided with synaptic elimination, expanded reproducibly during prolonged CPZ exposure, and incompletely resolved after toxin withdrawal, leaving a persistent smoldering microgliosis core. Together, these findings identify the SC as an MS-relevant site of injury and establish the CPZ-treated SC as a reproducible model for studying spatially patterned microglial reactivity, synaptic pathology, and incomplete inflammatory resolution after demyelinating injury.

## MATERIALS AND METHODS

### MATERIALS

#### Human Tissue

De-identified human postmortem brain specimens containing the SC were provided as non-human-subjects research/exempt under institutional policy. Samples were obtained from the University of Michigan Brain Bank (Michigan Medicine) and the National Institutes of Health (NIH) NeuroBioBank (Bethesda, MD, USA). The cohort comprised 9 donors total: 6 male donors and 3 female donors. Control tissue was obtained from 6 donors (5 male, 1 female) without diagnosed neurological disease. MS tissue was obtained from 3 donors (1 male, 2 female) with progressive MS. Donor characteristics are summarized in Table S1.

#### Mice

Male wild-type C57BL/6J mice (The Jackson Laboratory; stock no. 000664; Bar Harbor, ME, USA) were used for all experiments. Mice were housed on a 12-h light/12-h dark cycle with food and water *ad libitum* and were 8 weeks old at the start of experiments. All animal procedures were approved by the local Institutional Animal Care and Use Committee (IACUC) and were performed in accordance with institutional guidelines for animal welfare.

### METHODS

#### Cuprizone Mouse Model

Cuprizone-mediated demyelination was induced by feeding mice powdered rodent chow (LabDiet 5001; PicoLab) containing 0.2% (w/w) cuprizone (bis(cyclohexanone)oxaldihydrazone; Sigma-Aldrich, C9012-25G) for 6 or 9 weeks. Chow was provided *ad libitum* and replaced regularly throughout the experiment. CPZ chow was prepared fresh at each change by thoroughly mixing cuprizone powder into powdered ground chow. Control mice received the same powdered chow without CPZ. For recovery experiments, mice were treated with 0.2% CPZ for 6 weeks followed by return to standard rodent chow (CPZ withdrawal) for either 1 week or 3 weeks before tissue collection. During experimentally induced demyelination and recovery, the mice were monitored daily, and body weight was measured twice weekly.

#### Mouse tissue preparation and immunostaining

After diet administration, mice were euthanized with a lethal intraperitoneal dose of Avertin (2,2,2-tribromoethanol) and transcardially perfused with 0.1 M phosphate buffer (PB) followed by glyoxal fixative (9% glyoxal, 8% acetic acid in 0.1 M PB). Brains were dissected and post-fixed in the same glyoxal fixative for 4 h at 4°C, cryoprotected in 30% sucrose in 0.1 M PB at 4°C until equilibrated, embedded in a 1:1 mixture of 30% sucrose/0.1 M PB and O.C.T. compound (Thermo Fisher Scientific), and coronally cryosectioned at 10 μm for immunostaining. Sections were collected to sample the SC at three rostro–caudal levels: anterior (SC-A; Bregma − 3.15 to − 3.50), central (SC-C; Bregma − 3.51 to − 3.86), and posterior (SC-P; Bregma − 3.87 to − 4.22) (see Figure S2). Sections were blocked and permeabilized for 2 h at room temperature in 10% normal goat serum (MilliporeSigma) with 0.3% Triton X-100 (Sigma-Aldrich) in 0.1 M PB. For goat-derived primary antibodies, 10% normal donkey serum was used in place of goat serum. Primary antibodies were diluted in blocking solution and incubated overnight at room temperature: mouse anti-APP (clone 22C11; MilliporeSigma, MAB348; 1:200, RRID:AB_94882), rabbit anti-Caspr (abcam; ab34151, 1:1000, RRID:AB_869934), rat anti-CD45 (clone IBL-3/16; Bio-Rad, MCA1388; 1:100, RRID:AB_321729), rat anti-CD68 (clone FA-11; Bio-Rad, MCA1957; 1:1000, RRID:AB_322219), rabbit anti-cleaved caspase-3 (R&D Systems; MAB835, 1:1000, RRID:AB_2243951), rat anti-Clec7a (InvivoGen, mabg-mdect; 1:200, RRID:AB_2753143), mouse anti-MAG (clone 513; MilliporeSigma, MAB1567; 1:100, RRID:AB_11214010), chicken anti-MAP2 (EnCor, CPCA-MAP2; 1:1000, RRID:AB_2138173), chicken anti-NeuN (MilliporeSigma, ABN91; 1:1000, RRID:AB_11205760), rabbit anti-neurofilament 200 (MilliporeSigma, N4142; 1:1000, RRID:AB_477272), rabbit anti-PLP (clone EPR23504–106; Abcam, ab254363; 1:500, RRID:AB_3095302), rabbit anti-P2RY12 (AnaSpec, AS-55043A; 1:2000, RRID not available), and guinea pig anti-VGluT2 (Synaptic Systems, 135 404; 1:2000, RRID:AB_887884). After washing, sections were incubated with species-appropriate Alexa Fluor–conjugated secondary antibodies (Thermo Fisher Scientific) for 2 h at room temperature, washed, and mounted with Vectashield containing DAPI for nuclear counterstain (Vector Laboratories).

#### Human tissue preparation and immunostaining

Human postmortem brain tissue was dissected and formalin-fixed at autopsy. Tissue blocks containing the SC were identified, paraffin-embedded, and sectioned at 10 μm. For IHC, sections were deparaffinized and rehydrated through graded alcohols, using our established protocols [75]. Antigen retrieval was performed by heating slides for 10 min in Liberate Antibody Binding (L.A.B.) Solution (Polysciences) in a Coplin jar. Subsequent blocking, antibody incubations, washes, and mounting were performed as described for mouse tissue immunostaining. Myelin was detected using rat anti-MBP (clone 12; MilliporeSigma, MAB386, 1:500, RRID:AB_94975) and rabbit anti-PLP (clone EPR23504-106; Abcam, ab254363, 1:500, RRID:AB_3095302).

#### Quantification of myelin, axons, and microglia

Quantification of myelin (PLP, MAG, MBP), axonal/neuronal markers (NF200, MAP2), and microglial markers (P2RY12, Clec7a, CD45) was performed on coronal sections sampling three rostro–caudal SC planes (SC-A, SC-C, SC-P; Figure S2). For each animal, two sections per rostro–caudal plane were immunostained and imaged on a Zeiss LSM900 laser-scanning confocal microscope equipped with 405, 488, 561, and 640 nm lasers using ZEN Blue (v3.9; Zeiss). For each section, 20x tiled images spanning the entire SC were acquired, with acquisition settings held constant across samples within an experiment. Image analysis was performed in Fiji/ImageJ (NIH; v1.52k). Quantification was conducted blinded to experimental condition and followed published approaches with minor modifications [76]. Briefly, images were background-subtracted and thresholded using a single strategy per marker within an experiment. Thresholds were determined from representative images from each condition using the IsoData method (8-bit images; threshold range 85–255), and the same background subtraction and threshold parameters were then applied to all images for that marker within the experiment. Regions of interest (ROIs) corresponding to the whole SC and to superficial versus intermediate/deep layers of the SC were delineated using DAPI or relevant immunostained proteins (see above) together with reference to the mouse brain atlas. The HG compartment was defined as a contiguous area of elevated Clec7a signal, defined as the threshold above the mean of the whole SC in the same section after background subtraction. For each ROI, immunoreactivity was quantified as the percent area occupied by threshold-positive signal in the resulting binary mask using the ‘analyze particles’ function. Values from each hemisphere and both sections were averaged to generate a single value per animal for each rostro–caudal plane.

#### Synaptic density analysis

Density of synaptic inputs was assessed by quantifying VGluT2 immunoreactivity in sSC and idSC, and by comparing HG versus adjacent LG regions within each layer. For each animal, 1–2 non-overlapping 63x fields of view were acquired per hemisphere in each region (sSC-HG, sSC-LG, idSC-HG, idSC-LG) from two sections, yielding 6–8 fields of view per region per animal (1–2 fields × 4 hemispheres) and 12–16 fields of view per layer per animal. Images were collected on a Zeiss LSM900 laser-scanning confocal microscope using identical acquisition settings across groups within an experiment. For each field of view, a z-stack comprising three optical sections spaced at 1 μm was acquired, resulting in 18–24 single-plane images per region per animal (6–8 fields × 3 planes) and 48–96 single-plane images per animal across all four regions. Image analysis was performed blinded to experimental condition in Fiji/ImageJ. Single z-planes were background-subtracted and thresholded using a single thresholding strategy per experiment, as described above. Regions of interest were delineated for sSC and idSC based on DAPI morphology and atlas reference, and HG regions were defined by contiguous areas of high Clec7a immunoreactivity. VGluT2 signal was quantified as percent area occupied by threshold-positive puncta using the ‘analyze particles’ function. Values were averaged across z-planes and fields of view for each hemisphere and then across hemispheres to generate a single VGluT2 density value per animal for each region (sSC-HG, sSC-LG, idSC-HG, idSC-LG).

#### Cell, Axonal Damage, and Paranode Quantification

Cell densities were quantified for neurons (NeuN), infiltrating peripheral immune cells (CD45^high^), apoptotic cells (cleaved caspase-3), paranodes (CASPR), and axonal injury (APP). For each animal, single-plane 20x tiled images were acquired from two sections at each of three SC rostro–caudal levels (SC-A, SC-C, SC-P) using a Zeiss LSM900 laser-scanning confocal microscope with identical acquisition settings across groups within an experiment. Regions of interest corresponding to sSC and idSC were delineated based on DAPI morphology and atlas reference. Positive signals were manually counted in Fiji/ImageJ by an investigator blinded to treatment. For CD45^+^-monocytes, only CD45^high^, round cells consistent with infiltrating peripheral immune cells were counted, excluding CD45^low^ ramified microglia. Counts were normalized to ROI area to obtain densities (cells or APP^+^ counts per mm^2^). Values from both sections and both hemispheres were averaged to generate a single density value per animal for each rostro–caudal level and layer.

#### Microglial-Synapse Engulfment Analysis

Microglial synaptic engulfment was quantified as previously described [62, 63, 76]. Briefly, two SC-containing sections per mouse were immunostained for VGluT2, P2RY12, and CD68 and imaged on a Zeiss Observer spinning-disk confocal microscope equipped with 405, 488, 594, and 647 nm diode lasers using ZEN Blue software (Zeiss). Based on the gliosis mapping described above, high-magnification fields of view (63×) were selected per animal in HG regions and in adjacent low-gliosis LG regions. Within each field, microglial cells were imaged. Z-stacks were acquired with approximately 50 optical sections at 0.18 μm step size using identical acquisition settings across groups within an experiment. Image processing and quantification were performed blinded to treatment in Fiji/ImageJ and Imaris (Bitplane). Three-dimensional surfaces were generated for microglia (P2RY12) and lysosomes (CD68), and VGluT2 signal contained within CD68^+^ volumes inside microglial boundaries was quantified. For each animal, engulfment values were averaged to obtain compartment-specific measures for HG and LG regions within the SC.

#### Optomotor response assessment

Visual function was assessed in awake, freely moving mice one day prior to cuprizone administration (baseline) and after 6, 6 + 1, or 6 + 3 weeks of treatment. Visual acuity scores were measured using the OptoMotry system (CerebralMechanics) as previously described, with no prior training required [54, 76]. In brief, mice were placed on an elevated platform in the center of a four-walled chamber surrounded by computer monitors displaying visual stimuli. Animals were exposed to a virtual rotating cylinder of vertical sine-wave gratings (rotation speed 12°/s, 100% contrast) at varying spatial frequencies (cycles/degree), which elicit reflexive head-tracking movements when visible [54]. Behavior was recorded with an overhead camera and scored by a trained observer blinded to treatment. Visual acuity score was defined as the highest spatial frequency that reliably evoked head tracking and was determined by systematically increasing spatial frequency until the animal no longer responded.

#### HG overlap heatmap generation and alignment with known SC maps

To generate spatial overlap heatmaps of the HG compartment, Clec7a and P2RY12 immunostaining were performed on mouse SC sections and whole-SC tiled images were acquired. Tiled images were aligned to an anatomically matched coronal reference section from the Paxinos mouse brain atlas using the Fiji/ImageJ plug-in BigBrainWarp [50, 51]. After alignment, HG regions were delineated on the transformed images by manually tracing contiguous areas of elevated Clec7a signal, defined as the threshold above the mean of the whole SC in the same section after background subtraction. HG masks from individual animals were then exported and overlaid in a common atlas space to generate an overlap heatmap, in which each pixel value reflected the percentage of animals exhibiting HG at that location. Heatmaps were visualized as total extension (present in ≥ 1 animal), region present in most animals (> 75% overlap), and core region present in all animals (100% overlap).

#### Wind plot generation and analyses

Binary masks of HG compartments were exported from Fiji and analyzed in Python. For each mask, the outer boundary was extracted and represented as an ordered polygonal contour, which was then resampled to 360 points distributed evenly along the contour. To compare two compartments (e.g., across timepoints), we computed point-to-point correspondences by testing all circular shifts of the 360-point ordering and selecting the shift that minimized the overall boundary mismatch (summed Euclidean distances across all sets of points), thereby accounting for small indexing offsets introduced during contour sampling. Distances were then calculated between matched boundary points and converted from pixels to μm using the image scale. To obtain signed boundary change (expansion vs retraction), displacement vectors were projected onto the local outward direction defined by the centroid-to-boundary vector at each point (positive values indicate outward expansion; negative values indicate inward retraction). For visualization, signed distances were smoothed using a moving-average window of five points. Qualifications were performed on unsmoothed values.

#### Statistical analysis

Data are presented as mean ± standard error of the mean (SEM) from at least three independent biological replicates per group. Statistical analyses were performed in GraphPad Prism 10 and 11 (GraphPad Software). For comparisons between two groups, two-tailed unpaired or nested Student’s t-tests were used unless otherwise indicated. For within-animal repeated-measures comparisons (e.g., baseline versus post-treatment optomotor testing, or HG vs LG areas), two-tailed paired t-tests were used. For comparisons among multiple groups with a single independent variable, one-way ANOVA followed by Tukey’s multiple-comparisons test was used. For non-parametric comparisons among multiple groups, Kruskal-Wallis test was used. Non-significant results are reported as ns (p ≥ 0.05). Statistical significance was defined as *p* < 0.05 (*), p < 0.01 (**), p < 0.001 (***), and *p* < 0.0001 (****).

## RESULTS

### Postmortem MS Tissue Reveals Superior Colliculus Demyelination

**Figure 1:**
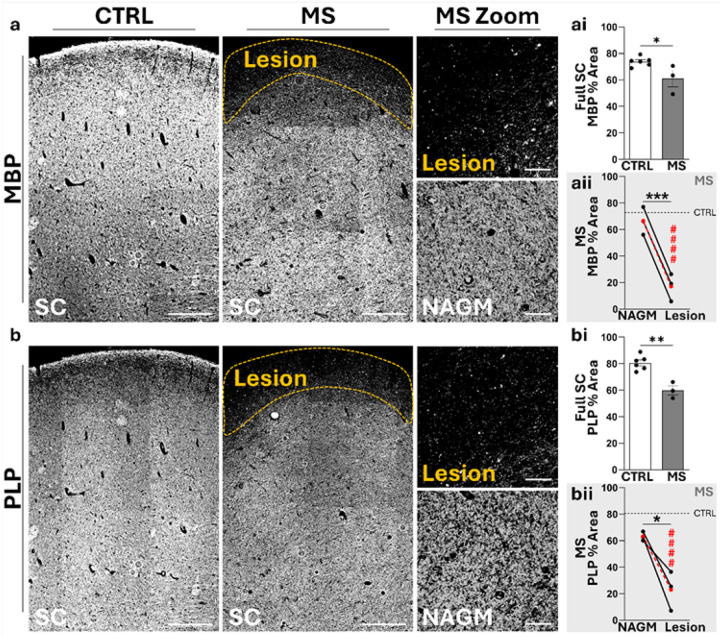
Demyelination in the Superior Colliculus of Postmortem Human MS Tissue. Representative overview images of coronal human postmortem superior colliculus (SC) sections from control donors (CTRL) without diagnosed neurological disease or people with MS, stained against (**a**) myelin basic protein (MBP) and (**b**) the myelin proteolipid protein (PLP). A distinct demyelinated lesion within the SC has been outlined (orange dashed line). Higher magnification images (MS Zoom) for both markers are shown to highlight detailed myelin structure in the lesion vs. normal-appearing gray matter (NAGM). Quantifications of (**ai**) MBP and (**bi**) PLP in the full SC show reduced myelin densities in the SC of MS tissue compared to CTRL. Within MS tissue, lesions show vastly reduced (**aii**) MBP^+^ and (**bii**) PLP^+^-myelin sheaths compared to control tissue or NAGM. Scale bars, overview image 500 μm, zoom image 100 μm. n = 3–6 subjects. Data represent mean ± SEM; red data points visualize average values for each designated group, significant differences with (*) p < 0.05, (**) p < 0.01, (***) p < 0.001. t-test, unpaired (**ai,bi**), paired or unpaired (**aii,bii**). #, denotes CTRL vs. NAGM or lesion (unpaired); *, denotes NAGM vs. lesion comparison (paired).

To first interrogate whether the SC is impacted in MS, we assessed myelin in postmortem SC from donors with MS compared to control specimens from donors without neurological disease (Fig. 1, Table S1). Confocal imaging of myelin basic protein (MBP, Fig. 1a) and myelin proteolipid protein (PLP, Fig. 1b) identified a loss of myelin in the SC in MS compared to controls (Fig. 1ai,bi). This reduction was most apparent in distinct demyelinated lesions in the SC, showing markedly lower myelin content compared to controls or normal-appearing gray matter (NAGM) in MS (Fig. 1aii,bii). These data are in line with previous work showing demyelination in other parts of the visual circuit in MS, including the optic nerve, lateral geniculate nucleus, optic radiations, and visual cortex [3, 8, 9, 19–21, 33, 34, 55, 57, 76, 81, 82]. Additionally, degeneration of the SC and the tectum of the midbrain has been documented in other neurological conditions such as progressive supranuclear palsy, Parkinson’s disease, and Lewy body dementia [6, 10, 12, 30, 41, 47, 48, 53, 59, 68, 70].

### Cuprizone Causes Visual Dysfunction and Widespread SC Demyelination while Preserving Neurons and Axons across SC Layers

**Figure 2:**
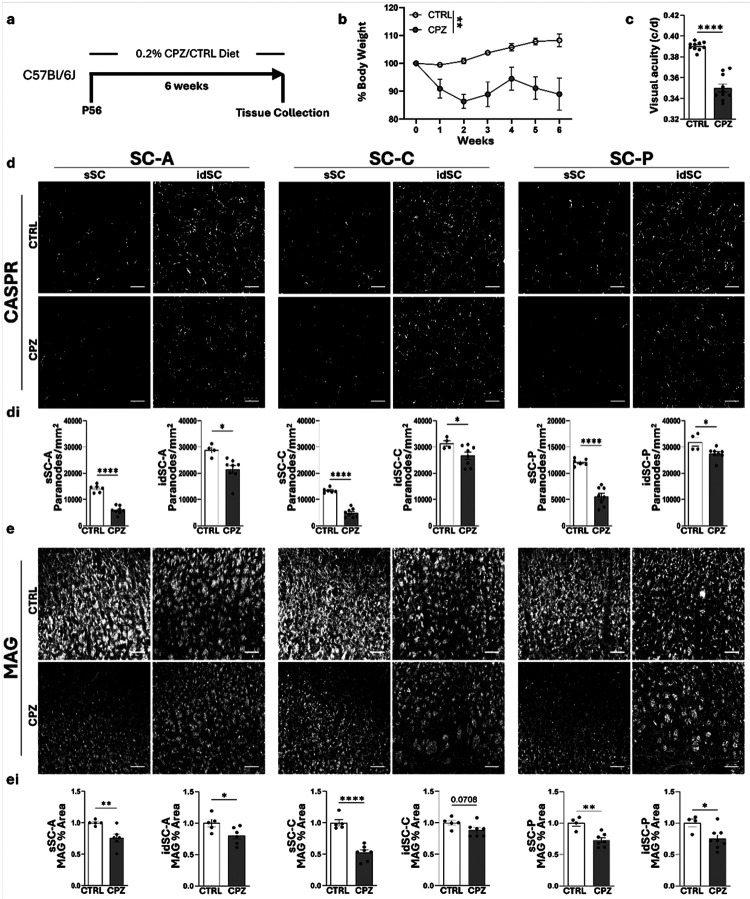
CPZ Treatment Induces Visual Dysfunction and Widespread Demyelination Across SC Layers and the Rostro–caudal Axis. (**a**) Schematic of the 6wk CPZ treatment experimental design. P56 mice were either fed 0.2% CPZ or a control diet for 6 weeks before tissue collection. (**b**) Body weight was measured weekly and plotted as % change relative to the starting weight at treatment start. CPZ-treated mice showed a mild reduction in body weight compared to CTRL. (**c**) Following six weeks of treatment, CPZ-treated mice exhibited significant visual impairment, evidenced by a marked reduction in visual acuity (cycles/degree) during optomotor testing. (**d-e**) Representative images of anterior SC (SC-A), central SC (SC-C), and posterior SC (SC-P) along the rostro–caudal axis in the superficial layers of the SC (sSC) and the intermediate and deep layers of the SC (idSC) from CTRL or CPZ-treated mice showing (**d**) the paranodal marker contactin-associated protein (CASPR) and (**e**) the myelin marker myelin-associated glycoprotein (MAG). Quantifications of (**di**) CASPR^+^-paranode count and (**ei**) MAG^+^-myelin density (normalized to CTRL) show widespread demyelination in the sSC and idSC across all rostro–caudal planes. Scale bars, 10 μm (**d**) and 40 μm (**e**). n = 4–8 mice. Data represent mean ± SEM; significant differences with (*) p < 0.05, (**) p < 0.01, (****) p < 0.0001. t-test, nested (**b**) and unpaired (**c-e**).

To provide a first comprehensive assessment of pathology in the SC in an MS-relevant setting, we utilized the CPZ mouse model of demyelinating disease, one of the most widely used preclinical models of MS [27, 66]. Mice were fed 0.2% (w/w) CPZ for 6 weeks—a commonly used regimen that produces robust gray matter demyelination (Fig. 2a) [24, 27, 35, 66, 67]—and compared to control mice on a standard rodent diet without CPZ. To maintain continuity with most CPZ paradigms, and to model the more severe MS disease course and greater disability accumulation prevalent in males with MS [7, 14, 25, 27, 66], we focused our analyses on male mice. As expected [27, 66], CPZ-treated mice exhibited a moderate reduction in body weight compared to controls (Fig. 2b). Optomotor testing also revealed a marked reduction in visual acuity following CPZ treatment (Fig. 2c), evidence for clear visual system dysfunction in these mice. We therefore next assessed hallmark MS pathologies, including demyelination, neuroaxonal degeneration, and inflammation within the SC.

In line with previous work describing mouse SC organization into defined layers [5, 11], we found different densities in myelinated axons at baseline, with intermediate/deep layers (idSC) showing relatively higher densities compared to the superficial layer (sSC) (Figure S1). Consequently, SC layers were analyzed separately in subsequent experiments. In addition, because CPZ pathology is spatially heterogeneous and has been reported to vary with anatomical level along the rostro–caudal (anterior–posterior) axis in other regions, most prominently described for the corpus callosum [69, 79, 80], we separately quantified pathology in anterior (SC-A, Bregma − 3.15 to −3.5), central (SC-C, Bregma − 3.51 to −3.86), and posterior (SC-P, Bregma − 3.87 to −4.22) coronal planes of the SC (Figure S2). We first interrogated changes in myelin by immunolabeling SC-A, SC-C, and SC-P sections for paranodal marker contactin-associated protein (CASPR, Fig. 2d) and myelin sheath proteins myelin-associated glycoprotein (MAG, Fig. 2e) and PLP (Figure S3a). Quantification of all markers revealed widespread demyelination across all rostro–caudal SC planes following CPZ compared to controls (Figs. 2di,ei and S3ai). Across planes, myelin loss appeared more significant in sSC than idSC (Figure S1), which may reflect layer-specific susceptibility or differences in baseline myelin density that can influence quantitative sensitivity and dynamic range in immunofluorescence-based measurements [74].

**Figure 3:**
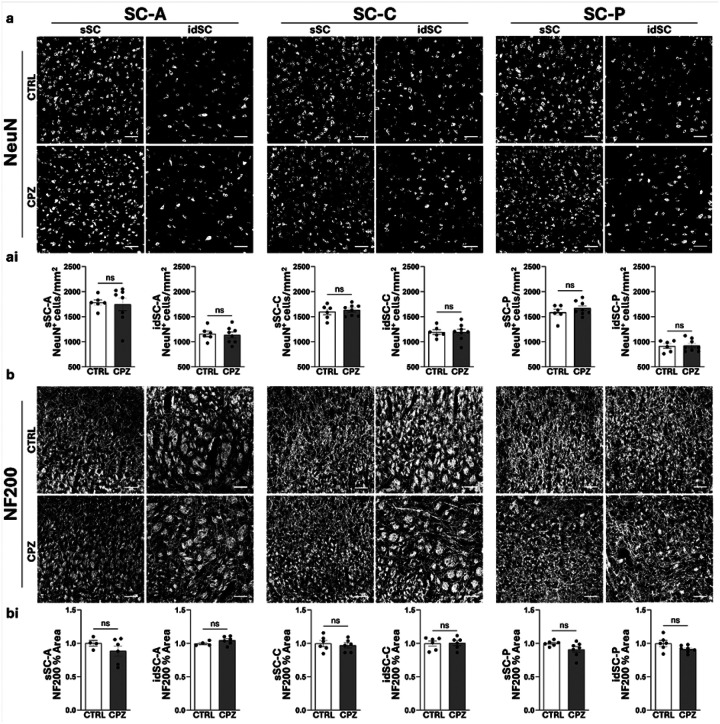
Neuronal and Axonal Densities Are Preserved Following CPZ Treatment. Analysis of adjacent SC-A, SC-C, and SC-P sections from the same mice as shown in Fig. 2. (**a-b**) Representative images along the rostro–caudal axis in the sSC and the idSC from CTRL or CPZ-treated mice showing (**a**) the neuronal cell marker neuronal nuclei (NeuN) and (**b**) the axonal marker neurofilament 200 (NF200). Quantifications of (**ai**) NeuN^+^-neuron counts and (**bi**) NF200^+^-axon density (normalized to CTRL) show no detectable change in neuroaxonal densities across the SC following CPZ treatment compared to CTRL. Scale bars, 40 μm. n = 4–8 mice. Data represent mean ± SEM; ns, non-significant results. t-test, unpaired.

We next tested whether pronounced demyelination leads to neuronal cell death or axonal degeneration in the SC of these mice. Quantification of immunolabeled neuronal nuclei (NeuN) and microtubule-associated protein 2 (MAP2, Figs. 3a and S3b) to assess neuronal density, and neurofilament 200 (NF200) (Fig. 3b) to assess axonal densities, revealed no detectable differences in either sSC or idSC layers of SC-A, SC-C, and SC-P sections following CPZ treatment compared to controls. To independently corroborate these findings, we also assessed cleaved caspase-3 and amyloid precursor protein (APP) in adjacent sections, revealing no detectable apoptotic cells or APP^+^- axonal pathology across the SC, respectively (Figure S4). Together, these results demonstrate that widespread demyelination occurs across SC layers and the rostro–caudal axis without the induction of neuronal cell death or axonal loss. These data are consistent with published work using comparable CPZ treatment regimens, showing pronounced demyelination across the brain with preserved neuroaxonal integrity [27, 66].

### Cuprizone induces a spatially stereotyped high-microgliosis compartment that does not correspond to canonical SC maps

Widespread CPZ-induced demyelination in other brain regions is typically accompanied by diffuse, pronounced local inflammation, with reactive microgliosis among the earliest detectable secondary responses to myelin injury [27, 66]. To determine whether broad SC demyelination is similarly associated with widespread microglial activation, we immunolabeled adjacent SC-A, SC-C, and SC-P sections for the homeostatic microglia marker purinergic receptor P2Y12 (P2RY12) and the disease-associated microglia marker Clec7a (Figure S5a,b). Quantification of confocal images revealed a robust reduction in P2RY12 immunoreactivity and a concomitant increase in Clec7a signal in CPZ-treated mice compared to controls, consistent with widespread reactive microgliosis and a shift away from a homeostatic state to a disease-associated phenotype (Figure S5ai,bi). These changes were present in both sSC and idSC layers and across SC-A, SC-C, and SC-P planes, indicating broad microglial activation throughout the SC along the rostro–caudal axis.

**Figure 4:**
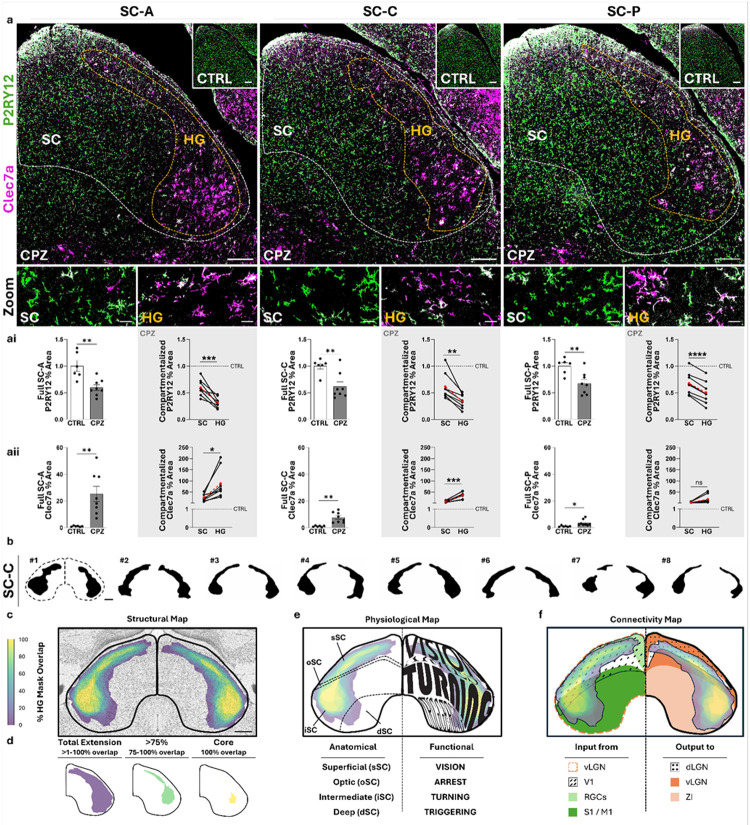
A Stereotyped High-Microgliosis Compartment Spans Parts of the sSC and idSC Without Aligning to Canonical SC Maps. Analysis of adjacent SC-A, SC-C, and SC-P sections from the same mice as shown in Figs. 2–3. (**a**) Representative tiled images of the entire SC along the rostro–caudal axis from CTRL (inset) or CPZ-treated mice showing the homeostatic microglial marker purinergic receptor P2Y12 (P2RY12, green) and the disease-associated microglial marker C-type lectin domain containing 7a (Clec7a, magenta). The SC is denoted by a white dashed line, and details are shown in zoom images. Quantifications across the full SC (SC) identified (**ai**) a reduction in P2RY12^+^-microglia density and (**aii**) an increase in Clec7a^+^-microglia density following CPZ treatment (inset, see also Figure S7d,e for detailed representative images) across all rostro–caudal planes (graphs on white background, normalized to CTRL). Closer inspection of full SC-tiles revealed a discrete high-microgliosis compartment (HG, orange outline) spanning parts of both the sSC and idSC in CPZ-animals. The HG compartment was defined as contiguous SC areas in which Clec7a signal exceeded the mean Clec7a intensity of the whole SC after background subtraction, showing especially pronounced alterations in both markers compared to the full SC within CPZ animals (graphs on gray background, normalized to CTRL). (**b**) Representative masks showing the shape and extension of SC-C HG regions within the SC (dashed black outline). For each CPZ-treated animal, these regions were delineated by manually tracing contiguous areas of Clec7a signal that exceeded the mean signal intensity of the total SC. Scale bar, 200 μm. (**c**) HG masks from individual animals were then used to generate an overlap heatmap, (**d**) distinguishing the total extension of the HG region (present in ≥1 animal), the region affected by HG in most animals (>75% overlap), and the core region affected by HG in all animals (100% overlap). Heatmap projection onto established (**e**) anatomical, functional, and (**f**) connectivity (SC inputs and outputs) maps of the SC, modified from[5, 11, 77], show no clear alignment with canonical SC structures or functional units. vLGN, ventral lateral geniculate nucleus (LGN); dLGN, dorsal LGN; V1, primary visual cortex; RGC, retinal ganglion cell; S1, primary somatosensory cortex; M1, primary motor cortex, ZI, zona incerta. Scale bars, 200 μm (**a_tiled_,b**) and 20 μm (**a_zoom_**). n = 6–8 mice. Data represent mean ± SEM; red data points visualize average values for each designated group, ns, non-significant results, significant differences with (*) p < 0.05, (**) p < 0.01, (***) p < 0.001, (****) p < 0.0001. t-test, unpaired (graphs on white background), paired (graphs on gray background).

Consistent with this, tiled images spanning the full SC corroborated P2RY12 downregulation and Clec7a induction across all rostro–caudal planes (Fig. 4ai,aii, full SC ctrl vs. CPZ). Despite this widespread response, closer inspection of full SC-tiles revealed a discrete high-microgliosis compartment (HG) in which microglial alterations were especially pronounced (Fig. 4a, orange outlines). This subregion was most clearly delineated by markedly elevated Clec7a relative to the remainder of the SC in CPZ-treated mice and showed concomitantly lower P2RY12 compared to other less inflamed SC regions (Fig. 4a, graphs on gray background, SC vs. HG). The HG compartment stereotypically spanned both sSC and idSC, comprising a large core within the ventro-lateral idSC with a slimmer lateral-to-medial neck region extending along the dorsal border of the sSC. Consistently, separate within-animal comparisons of high-versus low-microgliosis regions in each layer identified substantially larger changes in the idSC than the sSC (ΔP2RY12: −9.1% to − 23.8% in sSC vs. −34.5% to − 58.2% in idSC; ΔClec7a: 1.343- to 2.983-fold in sSC vs. 26.81- to 55.51-fold in idSC), and confirmed that heightened microgliosis occurred in subareas of both layers in CPZ-treated animals (Figure S6a,b).

Immunostaining for the pan-myeloid marker CD45 yielded consistent results, showing increased CD45 immunoreactivity in typically CD45^low^ microglia in CPZ-treated mice compared to controls, with a further elevation within the HG compartment (Figure S7a). CD45 staining also enabled assessment of infiltrating peripheral monocytes, characterized by high CD45 expression and round morphology, distinguishing them from CD45^low^, ramified microglia. In line with published work describing minimal to no peripheral immune cell recruitment in the CPZ model [27, 66], we observed no evidence for peripheral monocyte accumulation across SC layers or planes (Figure S7b), consistent with SC inflammation and HG compartment formation being primarily organized by CNS-intrinsic cues in this model.

Notably, the HG compartment was present in all CPZ-treated animals and appeared with strikingly consistent shape and location within the SC (Fig. 4b, see individual SC-C HG regions for all eight CPZ-treated animals). To map this compartment while accounting for potential sample distortion during tissue preparation, we used the Fiji plug-in BigBrainWarp to align full-SC confocal tiles to the corresponding coronal section from the Paxinos mouse brain atlas (Figure S6c) [50, 51]. Using these atlas-aligned masks, we generated an overlap heatmap of HG regions across animals, distinguishing the total extension of the affected region (present in ≥ 1 animal), the region affected in most animals (> 75% overlap), and the core region affected in all animals (100% overlap) (Fig. 4c,d). Projection of this heatmap onto established anatomical SC maps [5, 11, 77] showed partial yet incomplete overlap with superficial and intermediate/deep layers, including the myelin-rich optic layer, and thus no clear alignment with these anatomical boundaries (Fig. 4e, left). Likewise, comparison with proposed functional SC subdivisions [77], including areas relevant to vision, as well as arrest, turning, and triggering behaviors, revealed no clear correspondence (Fig. 4e, right). We also observed only limited overlap with known SC connectivity maps [5, 11], including, but not limited to, key inputs from retina, ventral LGN, and V1, S1, and M1 cortical areas, as well as relevant outputs to the dorsal and ventral LGN and zona incerta, without a clear match to specific input or output territories. Together, these analyses suggest that the characteristic shape and location of the HG compartment are not readily explained by selective vulnerability of currently defined anatomical, functional, or connectivity-based SC subregions.

### The High-Microgliosis Compartment Tracks Synaptic Elimination rather than Local Demyelination Severity

To assess whether differences in microglial state at baseline could explain HG compartmentalization after CPZ, we next projected the average > 75% HG region (present in the vast majority of CPZ-treated animals) onto BigBrainWarp-aligned tissue from matched control mice and assessed P2RY12 and Clec7a expression (Figure S6d,e). In controls, the projected LG and HG regions showed uniform microglial tiling with consistently high P2RY12 expression and largely absent Clec7a immunoreactivity (Figure S6di, ei), suggesting no baseline differences in these markers and no evidence for microglial compartmentalization within healthy animals.

**Figure 5:**
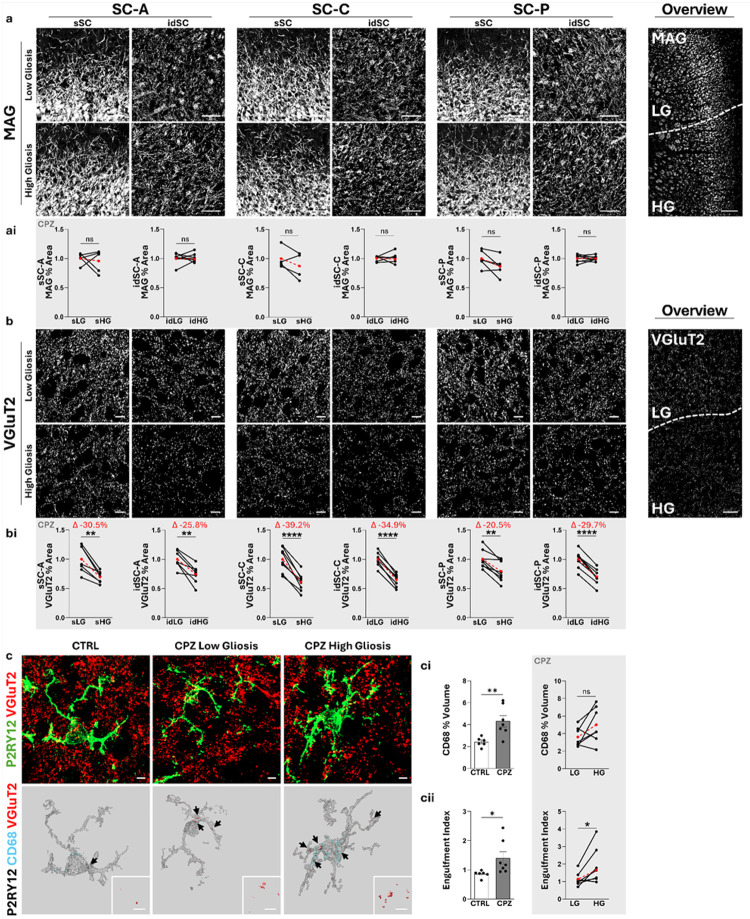
The HG Compartment Tracks Synaptic Loss and Microglial Engulfment Rather Than Local Demyelination Severity. Analysis of adjacent SC-A, SC-C, and SC-P sections from the same mice as shown in Figs. 2–4. (**a,b**) Representative images along the rostro–caudal axis from CPZ-treated mice in low-gliosis (LG) and high-gliosis (HG) regions of the sSC and idSC showing (**a**) MAG and (**b**) the synaptic marker vesicular glutamate transporter 2 (VGluT2). (**ai**) Comparative assessment of myelin density in superficial (s) or intermediate/deep (id) regions with low-gliosis (LG) or high-gliosis (HG) using MAG immunostaining revealed no differences (normalized to the LG region). (**bi**) Comparative assessment of synaptic density in sLG, sHG, idLG, and idHG regions using VGluT2 immunostaining revealed significantly reduced synaptic densities in HG areas across SC layers and the rostro–caudal axis (normalized to the LG region). Delta values represent the average % reduction in synaptic densities in HG areas compared to LG regions. (**a,b**) SC overview images illustrating no differences in MAG^+^-myelin densities but lower density of VGluT2^+^-synapses in HG compared to LG regions following CPZ. (**c**) Representative immunofluorescence images (top) and 3D surface rendering (bottom) of P2RY12^+^-microglia (top: green, bottom: gray) containing engulfed VGluT2^+^-synapses (red) within CD68^+^-microglial lysosomes (blue) from CTRL and CPZ-treated mice in LG and HG regions of the SC. Black arrows denote VGluT2^+^ signal within microglial lysosomes, and inset shows magnified engulfed synaptic material. Quantification of (**ci**) CD68 volume shows increased lysosomal content in microglia following CPZ compared to CTRL, with a trend toward further increased lysosomal content in HG areas compared to LG regions. Quantification of (**cii**) engulfed VGluT2 volume within microglial lysosomes relative to the total microglial volume (engulfment index) shows increased synaptic engulfment in microglia following CPZ compared to CTRL, with a further increase in engulfment in HG areas compared to LG regions. Graphs on a gray background show within CPZ comparisons. Scale bars, 50 μm (**a**) and 100 μm (overview), 10 μm (**b**) and 50 μm (overview), and 4 μm (**c**). n = 4–8 mice. Data represent mean ± SEM; red data points visualize average values for each designated group, ns, non-significant results, significant differences with (*) p < 0.05, (**) p < 0.01, (****) p < 0.0001. t-test, unpaired (graphs on white background), paired (graphs on gray background).

Because CPZ induces demyelination [27, 66], and our initial analyses were performed at the level of whole sSC and idSC layers, we next reanalyzed MAG and PLP immunoreactivity in HG and adjacent LG areas of CPZ-treated mice to test whether subregional differences in myelin loss might explain HG compartmentalization (Figs. 5a, S8a). Unexpectedly, quantification of MAG (Fig. 5ai) and PLP (Figure S8ai) revealed no differences in myelin between HG and LG within either layer, suggesting comparable myelin loss in HG versus LG regions after CPZ. Likewise, projection of the average > 75% HG region onto BigBrainWarp-aligned control tissue showed comparable baseline myelin levels across layers and rostro–caudal planes (Figure S8b). Together, these findings suggest that neither baseline myelin content nor the degree of CPZ-induced demyelination is sufficient to explain the observed compartmentalized HG microgliosis module in the SC.

An emerging, underappreciated pathology in MS and MS-relevant models is the disruption of neural circuits via the loss of synaptic terminals [31, 63, 73, 76]. Our previous work identified early synapse loss in the dorsal LGN as a functionally relevant pathology that can precede neuroaxonal degeneration across multiple preclinical MS models [76]. We therefore immunostained adjacent SC-A, SC-C, and SC-P sections against synaptic inputs using vesicular glutamate transporter 2 (VGluT2) (Fig. 5b), and observed markedly reduced VGluT2^+^-synaptic density in HG regions (average Δ−30.1%) of both sSC and idSC compared to adjacent LG regions of the same layers in CPZ-treated mice (Fig. 5bi). These differences were not explained by baseline variation, as the projected > 75% HG region in control tissue identified identical VGluT2^+^-synaptic density to adjacent projected LG regions across layers and rostro–caudal planes (Figure S8c). Thus, the degree of CPZ-induced secondary synaptic injury, rather than differences in primary demyelination per se, most closely aligned with the HG compartment across the SC (Figure S8d).

To further probe the relationship between compartmentalized microglial reactivity and synaptic loss, we next assessed microglia-mediated engulfment of VGluT2^+^-terminals (Fig. 5c). We co-immunolabeled microglia using P2RY12 and lysosomal marker CD68, together with VGluT2. Quantification revealed increased CD68^+^-phagolysosomes and a robust induction of microglial engulfment of VGluT2^+^-inputs in the SC of CPZ-treated mice compared to controls (Fig. 5ci), with VGluT2^+^-synapse engulfment further elevated within HG regions compared to adjacent LG areas (Fig. 5cii). Together, these data support a tight link between microglial reactivity and synaptic pathology that may govern the extent and spatial patterning of secondary injury subsequent to initial myelin loss.

### The High-Microgliosis Compartment Shows Spatially Stereotyped Expansion during Prolonged CPZ Exposure and Partial Resolution after Withdrawal

**Figure 6:**
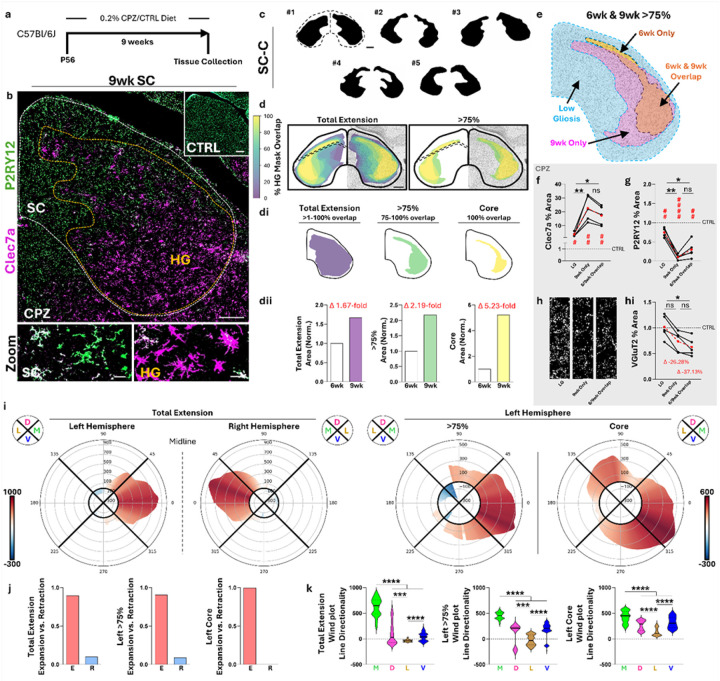
Prolonged CPZ Treatment Expands the HG Compartment and Worsens Synaptic Loss in a Stereotyped Manner. **(a)** Schematic of the 9wk CPZ treatment experimental design. P56 mice were either fed 0.2% CPZ or CTRL diet for 9 weeks before tissue collection. **(b)** Representative tiled image of the entire SC-C from CTRL (inset) or CPZ-treated mice stained against P2RY12 (green) and Clec7a (magenta), showing the high-microgliosis compartment (HG, orange outline) in CPZ-animals. The full SC is outlined with a white dashed line (SC), and details are shown in zoom images. **(c)** Representative masks showing the shape and extension of SC-C HG regions within the SC (dashed black outline) were generated for all animals as described in Fig. 4. Scale bar, 200 μm. **(d)** Representative HG mask overlap heatmaps, **(di)** distinguishing the total extension of the HG region (present in ≥1 animal), the region affected by HG in most animals (>75% overlap), and the core region affected by HG in all animals (100% overlap). **(dii)** Evaluations of total extension area (purple), >75% overlap area (green), and core region area (yellow) in 9wk CPZ HG mask overlap area compared to 6wk CPZ (normalized to 6wk CPZ). Delta values represent the fold increase in 9wk CPZ HG area compared to 6wk CPZ HG area. **(e)** Schematic to compare the 6wk CPZ >75% heatmap area (orange) to the 9wk CPZ >75% heatmap area (pink). The area shown in blue comprises the LG region of the SC. **(f,g)** Subregional quantification of **(f)** Clec7a and **(g)** P2RY12 within CPZ animals identified comparable alterations in Clec7a^+^- and (g) P2RY12^+^-microglia densities in newly emerging HG regions (9wk Only) and sustained HG areas (6/9wk Overlap) compared to CTRL (normalized to CTRL). **(h)** Representative images from CPZ-treated mice of VGluT2 in LG, 9wk Only, and 6/9wk Overlap regions. **(hi)** Quantification shows a reduction in VGluT2^+^-synapse density in the 6/9wk Overlap region compared to the LG region (normalized to CTRL). Delta changes represent average % reduction in synaptic densities in 9wk Only (Δ−26.28%) and 6/9wk Overlap (Δ−37.13%) HG regions compared to the LG region. **(i)** Wind plots comparing 6wk CPZ-treated animals to the 9wk CPZ-treated cohort represent the directionality and changes (μm) in the total extension (both hemispheres), the >75% area (left hemisphere), and the core (left hemisphere). **(j)** Evaluations of wind plots show a predominant expansion (E, red) of the total extension, the >75% area, and the core, with minimal to no retracted areas (R) in 9wk CPZ compared to 6wk CPZ. **(k)** Quantification of expansion directionality binned into medial (green, 316–45°), dorsal (pink, 46–135°), lateral (gold, 136–225°), and ventral (blue, 226–315°) quadrants, shows predominant expansion towards the brain midline with dorsal and ventral growth of the total extension, the >75% area, and the core. Scale bars, 200 μm (**b_tiled_,c,d**), 20 μm (**b_zoom_**), 5 μm (**h**). n = 3–5 mice. Data represent mean ± SEM; red data points visualize average values for each designated group; ns, non-significant results, significant differences with (*) p < 0.05, (**) p < 0.01, (***) p < 0.001, (****) p < 0.0001. One-way ANOVA **(f-h)**, Kruskal-Wallis test **(k)**. #, denotes statistical comparison to control; *, denotes statistical comparison within CPZ animals.

To interrogate the dynamics of HG compartment formation, we extended CPZ treatment in a separate cohort of mice to 9 weeks (9wk) (Fig. 6a). Given the high consistency of HG across rostro–caudal planes in the 6-week cohort, we focused subsequent analyses on central SC planes (Figure S2). After 9 weeks of CPZ, we immunolabeled microglia using P2RY12 and Clec7a (Fig. 6b), delineated HG regions in each animal (Fig. 6c), and generated overlap heatmaps (Fig. 6d) defining the total extension (present in ≥ 1 animal), the region present in most animals (> 75% overlap), and the core region present in all animals (100% overlap). Similar to 6 weeks, the HG compartment at 9 weeks exhibited a strikingly consistent shape and location, spanning both sSC and idSC and comprising a bulky core in the ventro-lateral-to-medial idSC with a substantial lateral-to-medial neck region extending along the dorsal sSC (Fig. 6c,d). Compared to 6 weeks, the 9-week cohort showed a marked expansion of the HG compartment (total extension: 1.67-fold, > 75% overlap: 2.19-fold, core: 5.23-fold) (Fig. 6dii), suggesting that not only the initial shape and localization of the HG compartment but also its expansion after prolonged CPZ exposure is highly stereotyped.

To validate and characterize this expansion, we projected the 6- and 9-week atlas-aligned > 75% HG masks onto 9-week tissue to distinguish newly emerged HG regions (9-week only), HG regions present at 6 weeks that persisted at 9 weeks (6/9-week overlap), and remaining LG tissue (Fig. 6e). Quantification of Clec7a (Fig. 6f) and P2RY12 (Fig. 6g) confirmed heightened microgliosis in newly emerged regions (9-week only) relative to LG areas, with sustained gliosis in the persistent HG compartment (6/9-week overlap). In line with HG tracking secondary synaptic pathology, VGluT2^+^-synaptic density was reduced by 26.28% in newly emerged HG regions compared with LG areas—similar to the average reduction observed at 6 weeks (Δ − 30.1%)—whereas HG regions already present at 6 weeks and maintained through 9 weeks exhibited a larger decrease (Δ − 37.13%), consistent with cumulative synaptic injury during prolonged CPZ exposure (Fig. 6h).

To further characterize HG expansion, we generated wind plots for both brain hemispheres, comparing 9-week versus 6-week cohorts (Fig. 6i,j). This analysis revealed a consistent and highly directed extension of the total HG compartment—originating from the ventro-lateral idSC core and dorsal sSC neck—predominantly toward the brain midline (Fig. 6k). Directionality analyses also suggested that the > 75% HG and core regions expanded preferentially medially, with additional dorsal and ventral spread, consistent with growth from a stable core rather than emergence from multiple independent foci. Of note, lateral expansion was more limited, likely constrained by the proximity of the HG compartment to the lateral SC tissue border.

**Figure 7:**
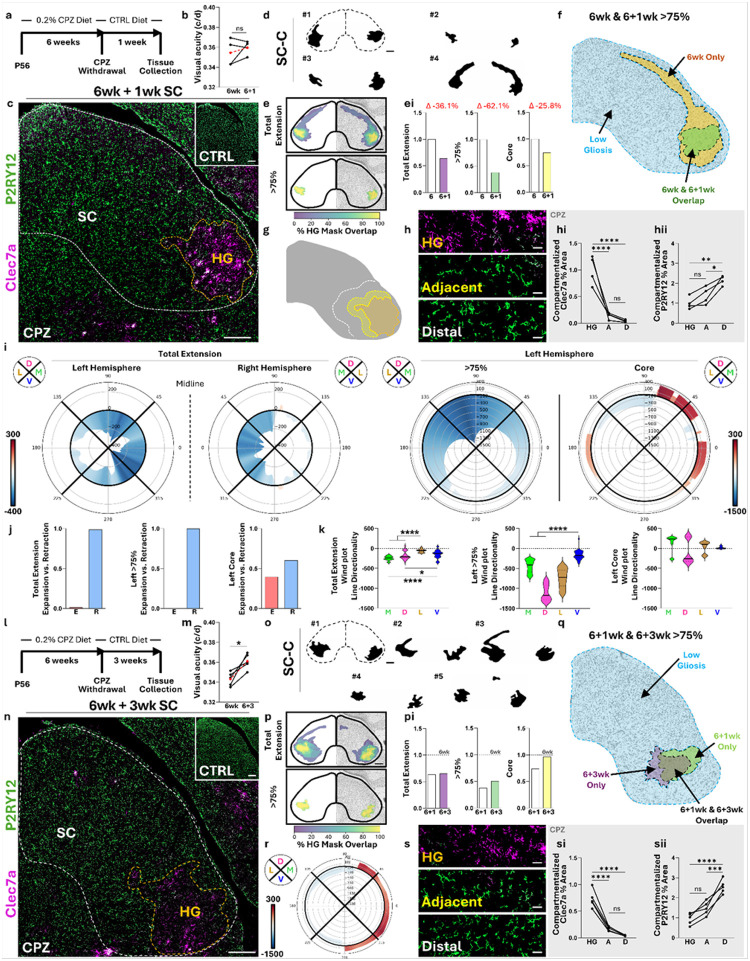
CPZ Withdrawal Drives Spatially Ordered Resolution of the HG Compartment With a Persistent Smoldering Core. (**a**) Schematic of the 6wk CPZ followed by 1wk on CTRL diet (6+1) experimental design. P56 mice were either fed 0.2% CPZ or CTRL diet for 6 weeks, followed by 1 week of recovery on CTRL diet. (**b**) Compared to 6wk CPZ, animals recovered for 1 week (6+1), showed no recovery of visual acuity at this early withdrawal timepoint. (**c**) Representative tiled image of the entire SC-C from CTRL (inset) or CPZ-treated mice stained against P2RY12 (green) and Clec7a (magenta), showing the high-microgliosis compartment (HG, orange outline) in CPZ-animals. The full SC is outlined by a white dashed line (SC). (**d**) Representative masks showing the shape and extension of SC-C HG regions within the SC (dashed black outline) were generated for each animal as described in Fig. 4. Scale bar, 200 μm. (**e**) Representative HG mask overlap heatmaps, distinguishing the total extension of the HG region (present in ≥1 animal) and the region affected by HG in most animals (>75% overlap). (**ei**) Evaluations of total extension area (purple), >75% overlap area (green), and core region area (yellow) in 6+1 HG mask overlap area compared to 6wk CPZ (normalized to 6wk CPZ). Delta values represent the percent decrease in 6+1 HG area compared to 6wk CPZ HG area. (**f**) Schematic to compare the 6wk CPZ >75% heatmap area (orange) to the 6+1 >75% heatmap area (green). The area shown in blue comprises the LG region of the SC. (**g**) Illustration of 6+1 analysis strategy to gradually measure microgliosis with increasing distance from the HG region. (**h-hii**) Representative images and quantification of Clec7a and P2RY12 across the remaining HG region, adjacent areas (0–400μm), and distal tissue (>400μm away from the HG region), show a sustained high Clec7a and low P2RY12 in the HG core with graded normalization with increasing distance from the core (normalized to the 6+1 HG region). (**i**) Wind plots comparing changes from 6wk CPZ-treated animals compared to the 6+1-treated cohort represent the directionality and changes (μm) in the total extension (both hemispheres), the >75% area (left hemisphere), and the core (left hemisphere). (**j**) Evaluations of wind plots show a predominant retraction (blue) of the total extension and the >75% area, with no expanded areas in 6+1 compared to 6wk CPZ. Whereas the core showed little net change and no clear pattern of expansion or retraction. (**k**) Quantification of expansion directionality binned into medial (green, 316–45°), dorsal (pink, 46–135°), lateral (gold, 136–225°), and ventral (blue, 226–315°) quadrants shows predominantly medial and dorsal retraction of the total extension and the >75% area, with no clear preference and only minimal changes of the core. (**l**) Schematic of the 6wk CPZ followed by 3wk on CTRL diet (6+3) experimental design. P56 mice were either fed 0.2% CPZ or CTRL diet for 6 weeks, followed by 3 weeks of recovery on CTRL diet. (**m**) Compared to 6wk CPZ, animals recovered for 3 weeks (6+3), showed recovery of visual acuity at the later withdrawal timepoint. (**n**) Representative tiled image of the entire SC-C from CTRL (inset) or CPZ-treated mice stained against P2RY12 (green) and Clec7a (magenta), showing the high-microgliosis compartment (HG, orange outline) in CPZ-animals. The full SC is outlined with a white dashed line (SC). (**o**) Representative masks showing the shape and extension of SC-C HG regions within the SC (dashed black outline) were generated as described in Fig. 4. Scale bar, 200 μm. (**p**) Representative HG mask overlap heatmaps, distinguishing the total extension of the HG region (present in ≥1 animal) and the region affected by HG in most animals (>75% overlap). (**pi**) Evaluations of total extension area (purple), >75% overlap area (green), and core region area (yellow) in 6+3 HG mask overlap area compared to 6+1 (normalized to 6wk CPZ). (**q**) Schematic to compare the 6+1 >75% heatmap area (green) to the 6+3 >75% heatmap area (purple). The area shown in blue comprises the LG region of the SC. (**r**) Wind plots comparing changes from 6+1-treated animals compared to the 6+3-treated cohort represent the directionality and changes (μm) in the >75% area (left hemisphere). (**s-sii**) Representative images and quantification of Clec7a and P2RY12 across the remaining HG region, adjacent areas (0–400μm), and distal tissue (>400μm away from the HG region), show a sustained high Clec7a and low P2RY12 in the HG core with graded normalization with increasing distance from the core (normalized to the 6+1 HG region). Scale bars, 200 μm (**c-e,k-m**) and 20 μm (**gi,p**). n = 4–5 mice. Data represent mean ± SEM; red data points visualize average values for each designated group; ns, non-significant results, significant differences with (*) p < 0.05, (**) p < 0.01, (***) p < 0.001, and (****) p < 0.0001. t-test, paired (**b,j**), one-way ANOVA (graphs on gray background), Kruskal-Wallis test (**hii**).

Because the CPZ model is widely used to study repair after toxin withdrawal [27, 66], we next asked whether HG microgliosis resolves in a stereotyped manner after CPZ cessation. We treated an additional cohort with CPZ for 6 weeks, followed by 1 week of CPZ withdrawal (6 + 1-week), allowing for recovery (Fig. 7a). After 1 week of withdrawal, mice showed no further decline in visual acuity but also no recovery at this early repair timepoint (Fig. 7b). We immunolabeled microglia using P2RY12 and Clec7a (Fig. 7c), delineated HG regions for each animal (Fig. 7d), and generated overlap heatmaps (Fig. 7e) as above. Following 6 weeks CPZ + 1 week recovery (6 + 1-week), the HG compartment remained highly stereotyped but was largely restricted to the ventro-lateral idSC, with a persistent core and loss of the dorsal sSC neck region in all but one animal (Fig. 7d). Compared to 6-week CPZ, heatmaps showed a pronounced reduction in HG extent (total: Δ − 36.1%; >75% overlap: Δ − 62.1%; core: Δ − 25.8%; Fig. 7ei). Together, these data suggest that HG resolution is also spatially patterned. To validate this, we projected the 6-week and 6 + 1-week atlas-aligned > 75% HG masks onto 6 + 1-week tissue to distinguish resolved HG territory (6-week only) from persistent HG regions (6/6 + 1-week overlap)(Fig. 7f). Quantification of Clec7a and P2RY12 across the remaining HG core, adjacent peri-core regions, and distal tissue (Fig. 7g) revealed sustained high Clec7a and low P2RY12 in the core with graded normalization with increasing distance from the core, consistent with an “outside-in” resolution pattern of microgliosis (Fig. 7h). Wind-plot analyses comparing 6 + 1-week to 6-week cohorts (Fig. 7i) showed robust reduction of the total and > 75% HG territories preferentially across medial and dorsal directions, whereas the core exhibited comparatively little net change and mixed local expansion and retraction (Fig. 7j,k), consistent with a relative persistence of the core during early recovery.

To test whether the remaining HG core resolves with longer recovery, we treated a final cohort of mice with 6 weeks of CPZ followed by 3 weeks of recovery (6 + 3 weeks) (Fig. 7l). These mice showed improved visual acuity compared to 6-week CPZ mice (Fig. 7m). As before, we again immunolabeled microglia using P2RY12 and Clec7a (Fig. 7n), delineated HG regions (Fig. 7o), and generated overlap heatmaps (Fig. 7p). As in the 6 + 1-week cohort, the residual HG compartment remained stereotyped and largely confined to the ventro-lateral idSC, persisting as a prominent core across animals (Fig. 7p). Comparison of HG extent between 6 + 3-week and 6 + 1-week cohorts revealed no further reduction in total, > 75% overlap, or core regions (Fig. 7pi–r). Consistently, quantification of Clec7a and P2RY12 across core, adjacent peri-core, and distal regions (Fig. 7s) confirmed sustained high Clec7a and low P2RY12 in the core with graded normalization with distance, suggesting persistent smoldering microgliosis centered around a stable HG core even after prolonged CPZ cessation.

## DISCUSSION

Here, we identify the SC as an MS-relevant site of gray matter injury by showing that it can harbor demyelination in MS, including focal lesions, thereby extending MS visual system pathology to an understudied visual and visuomotor hub [5, 6, 11, 77]. Although the MS cohort was small, the clear presence of SC demyelination supports disease relevance and motivated the use of CPZ-treated mice to dissect spatially organized pathological responses to demyelinating injury in the SC. In this model, we identified a highly reproducible microgliosis module with elevated reactivity within the SC. This HG compartment exhibits striking spatiotemporal stereotypy, including reproducible expansion and incomplete resolution, without conforming to established anatomical, functional, or connectivity-based SC maps. As such, the CPZ-treated SC provides a novel, tractable *in vivo* framework to interrogate how local tissue cues and cellular interactions regulate the induction, propagation, persistence, and resolution of spatially organized microglial reactivity in MS-relevant gray matter pathology.

Although CPZ does not model the full autoimmune and peripheral immune components of MS [27, 66], this paradigm provides a controlled system to dissect CNS-intrinsic cellular responses and secondary pathology following demyelinating injury. Consistent with extensive CPZ literature showing robust and temporally defined demyelination across white and gray matter regions, including the corpus callosum, cortex, hippocampus, and subcortical sensory networks [24, 27, 35, 66, 67, 69, 79, 80], we found widespread SC demyelination across superficial and intermediate/deep layers and along the rostro–caudal axis. These findings extend the spatial map of CPZ pathology to the SC and indicate that it is broadly susceptible to CPZ-induced myelin loss. Consistent with the oligodendrocyte-targeted nature of CPZ toxicity and similar to other brain regions [27, 66], SC demyelination occurred without detectable reductions in neuronal and axonal densities or evidence of increased apoptotic cells or axonal pathology.

Furthermore, in line with the well-established microglial response to CPZ-induced demyelination [27, 66], diffuse myelin loss was accompanied by widespread microgliosis across the SC, reflected by markedly reduced P2RY12 and increased Clec7a immunoreactivity across layers and rostro–caudal planes. However, despite this pattern of widespread microgliosis known to accompany broad diffuse demyelination, our data reveal an additional unexpected level of organization, showing the stereotyped emergence of a microglial module with heightened reactivity, which appeared in a consistent location across animals and spanned portions of both superficial and intermediate/deep SC layers. Atlas-aligned overlap heatmap projections of HG regions onto established SC maps showed no clear correspondence with canonical anatomical, functional, or input–output maps [5, 11, 77]. Baseline projection analyses also did not reveal pre-existing differences in the microglial markers, and HG versus adjacent LG regions showed comparable myelin densities after CPZ. Because these analyses rely on selected markers, they define regional differences in microglial reactivity rather than the full molecular diversity of microglial states within HG and LG regions. Nevertheless, these findings argue that HG compartmentalization is not readily explained by baseline microglial marker heterogeneity, local myelin architecture, or demyelination severity alone. Instead, it appears to represent a reproducible spatial module of heightened microglial reactivity shaped by disease-induced local tissue context.

Consistent with this interpretation, a defining feature of the HG compartment was its association with synaptic pathology. While myelin measures were comparable between HG and adjacent LG regions, VGluT2^+^-synaptic density was markedly reduced within HG territories, with more pronounced loss in persistent HG regions during prolonged CPZ treatment. CPZ also increased CD68^+^-phagolysosomal content and enhanced engulfment of VGluT2^+^-synaptic material, with engulfment further elevated in HG regions. In line with previous findings [13, 17, 28, 32, 45, 46, 71, 76], these results extend evidence that synaptic pathology can emerge early in MS-relevant settings and may contribute to circuit dysfunction before overt neuroaxonal loss. However, the present data do not establish whether microglial reactivity causes synaptic loss, whether synaptic injury enhances microglial reactivity, or whether both reflect a shared upstream cue independent from the degree of demyelination. Rather, they identify a tight spatial coupling between microglial reactivity and synaptic injury within a reproducible gray matter compartment. To our knowledge, such a spatially discrete microgliosis module dissociated from the degree of demyelination has not previously been described in the CPZ model, providing a robust framework to test in future work whether microglial phagocytic pathways, synaptic opsonization, or synapse-protective interventions modify HG-associated synaptic loss or compartment formation [28, 45, 76].

The temporal behavior of the HG compartment further supports its interpretation as a microgliosis module with stereotyped dynamics. With prolonged CPZ exposure, HG regions expanded reproducibly from a stable ventro-lateral core into adjacent territories rather than appearing as independent inflammatory foci. After CPZ withdrawal, HG regions partially contracted in a spatially ordered manner, with apparent normalization beginning at the periphery while a residual core persisted. This smoldering core suggests that reactivity can remain spatially restricted and sustained after cessation of the initiating demyelinating insult. Although this should not be directly equated with chronic active MS lesions [27, 66], it provides a tractable model to study CNS-intrinsic mechanisms of microglial reactivity propagation and incomplete resolution, processes relevant to progressive MS and other neurodegenerative diseases [9, 17, 56, 78]. This is of particular interest because microglia are key contributors to MS pathology and disability progression, and they adopt diverse reactive states that can influence local injury, repair, and circuit function [1, 29, 64]. These reactive states are believed to arise from the integration of heterogeneous local molecular cues and cellular interactions, yet their spatiotemporal distribution in most models can vary markedly across animals and even within a single brain region, making where and when they occur difficult to predict and complicating *in situ* mechanistic dissection. In this context, the reproducibility of the HG compartment creates an opportunity for future studies to dissect local determinants of microglial reactivity with unusual spatial precision. The clear association with VGluT2^+^ synaptic loss suggests that local synaptic activity, terminal-specific vulnerability, or synaptic tagging pathways may help define HG territories [13, 17, 28, 32, 45, 46, 71, 76]. Astrocyte–microglia interactions, localized inflammatory cues, cellular stress, myelin debris handling, and extracellular matrix alterations may also shape regional susceptibility and persistence [9, 27, 28, 45, 66, 76, 78]. Future studies using the CPZ-treated SC to compare HG and LG regions through spatial transcriptomics or proteomics, circuit tracing, and targeted microglial perturbation should help identify the cues and cellular partners governing HG induction, expansion, persistence, and resolution.

The discovery of a distinct HG compartment spanning the superficial SC layers also makes the CPZ-treated SC an exceptional target for future *in vivo* brain imaging. Because HG and LG territories occur in direct proximity within the same functional circuit, posterior cranial-window approaches could enable comparative assessment of microglial behavior, synaptic remodeling, and circuit integrity across adjacent HG vs. LG microenvironments without tissue aspiration or penetrating lens implantation[37, 38, 58]. Combined with sparse neuronal or synaptic labeling, this approach could help define how focal microglial reactivity relates to local synaptic injury and circuit remodeling over time. As a laminated midbrain structure, the SC also offers a distinct neuroanatomical context compared to the typically investigated neocortex, providing a platform to complement deep imaging work on cortical pathologies in MS.

We also identified impaired optomotor response in CPZ-treated mice, followed by improvement after prolonged CPZ withdrawal. Prior studies using acute dLGN slice recordings and visual evoked potentials have shown that CPZ disrupts visual system function, including impaired subcortical visual circuit activity, delayed visual conduction, and reduced visual evoked potential amplitudes during demyelination and recovery [2, 40, 60, 61, 72]. Our findings extend this work by demonstrating impairment of the optomotor response, a non-invasive, training-free behavioral measure of visual function [54]. However, because the optomotor response depends on distributed visual-motion and visuomotor pathways, including the accessory optic system and downstream motor circuits [16, 26, 54, 65], it serves as a useful behavioral complement to electrophysiological readouts of integrated visual and visuomotor dysfunction in CPZ-treated mice rather than a region-specific functional measure.

## CONCLUSIONS

Together, these findings establish the SC as an MS-relevant structure and identify a previously unrecognized, spatially stereotyped module of microglial reactivity within a defined visual circuit. The HG compartment shows that microgliosis after demyelinating injury is not necessarily diffuse or proportional to local myelin loss, but can organize into a reproducible territory that tracks synaptic pathology and exhibits stereotyped expansion and incomplete resolution. By enabling spatially precise comparisons of regions that differ in microglial reactivity, synaptic integrity, and recovery dynamics within the same circuit, this model provides a platform to define how local cues and cellular interactions govern the induction, propagation, persistence, and incomplete resolution of microglial reactivity. More broadly, these data support the concept that spatially organized microglia–synapse interactions may shape early secondary circuit injury and offer insight into persistent innate immune mechanisms relevant to MS and related neurodegenerative diseases.

## Supplementary Material

Supplementary Files

This is a list of supplementary files associated with this preprint. Click to download.

- 260522McGrathetalSupplementaryFigures.pdf

## Data Availability

All data supporting the findings of this study are included in the manuscript and supplementary materials. Additional raw data and analysis files are available from the corresponding author upon request. Custom code used for data analysis is available at: https://github.com/dfhannum/SC_ImageProcessing

## Abbreviations

ANOVA: analysis of variance
APP: amyloid precursor protein
CASPR: contactin-associated protein 1
cCasp3: cleaved caspase-3
CD45: cluster of differentiation 45
CD68: cluster of differentiation 68
Clec7a: C-type lectin domain containing 7a
CNS: central nervous system
CPZ: cuprizone
CTRL: control
DAPI: 4′,6-diamidino-2-phenylindole
dLGN: dorsal lateral geniculate nucleus
EAE: experimental autoimmune encephalomyelitis
HG: high-gliosis compartment/region
IACUC: Institutional Animal Care and Use Committee
idSC: intermediate/deep superior colliculus
IHC: immunohistochemistry
L.A.B.: Liberate Antibody Binding solution
LG: low-gliosis region
LGN: lateral geniculate nucleus
MAG: myelin-associated glycoprotein
MAP2: microtubule-associated protein 2
MBP: myelin basic protein
M1: primary motor cortex
MS: multiple sclerosis
NAGM: normal-appearing gray matter
NeuN: neuronal nuclei
NF200: neurofilament 200
NIH: National Institutes of Health
OMR: optomotor response
O.C.T.: optimal cutting temperature compound
PB: phosphate buffer
PLP: proteolipid protein
P2RY12: purinergic receptor P2Y12
RGC: retinal ganglion cell
ROI: region of interest
S1: primary somatosensory cortex
SC: superior colliculus
SC-A: anterior superior colliculus plane
SC-C: central superior colliculus plane
SC-P: posterior superior colliculus plane
SEM: standard error of the mean
sSC: superficial superior colliculus
V1: primary visual cortex
VGluT2: vesicular glutamate transporter 2
vLGN: ventral lateral geniculate nucleus
ZI: zona incerta
